# What can patients tell us about the quality and safety of hospital care? Findings from a UK multicentre survey study

**DOI:** 10.1136/bmjqs-2017-006974

**Published:** 2018-03-15

**Authors:** Jane K O’Hara, Caroline Reynolds, Sally Moore, Gerry Armitage, Laura Sheard, Claire Marsh, Ian Watt, John Wright, Rebecca Lawton

**Affiliations:** 1 Leeds Institute of Medical Education, University of Leeds, Leeds, UK; 2 Department of Quality and Safety, Bradford Institute for Health Research, Bradford, UK; 3 School of Health, University of Bradford, Bradford, UK; 4 Bradford Institute for Health Research, Bradford, UK; 5 Department of Health Sciences, University of York, York, UK; 6 Department of Epidemiology and Public Health, Royal Infirmary Bradford, Bradford, UK; 7 Institute of Psychological Sciences, University of Leeds, Leeds, UK

**Keywords:** adverse events, epidemiology and detection, human factors, medical error, measurement/epidemiology, patient safety, quality measurement

## Abstract

**Background:**

Patient safety measurement remains a global challenge. Patients are an important but neglected source of learning; however, little is known about what patients can add to our understanding of safety. We sought to understand the incidence and nature of patient-reported safety concerns in hospital.

**Methods:**

Feedback about the experience of safety within hospital was gathered from 2471 inpatients as part of a multicentre, waitlist cluster randomised controlled trial of an intervention, undertaken within 33 wards across three English NHS Trusts, between May 2013 and September 2014. Patient volunteers, supported by researchers, developed a classification framework of patient-reported safety concerns from a random sample of 231 reports. All reports were then classified using the patient-developed categories. Following this, all patient-reported safety concerns underwent a two-stage clinical review process for identification of patient safety incidents.

**Results:**

Of the 2471 inpatients recruited, 579 provided 1155 patient-reported incident reports. 14 categories were developed for classification of reports, with communication the most frequently occurring (22%), followed by staffing issues (13%) and problems with the care environment (12%). 406 of the total 1155 patient incident reports (35%) were classified by clinicians as a patient safety incident according to the standard definition. 1 in 10 patients (264 patients) identified a patient safety incident, with medication errors the most frequently reported incident.

**Conclusions:**

Our findings suggest that patients can provide insight about safety that complements existing patient safety measurement, with a frequency of reported patient safety incidents that is similar to those obtained via case note review. However, patients provide a unique perspective about hospital safety which differs from and adds to current definitions of patient safety incidents.

**Trial registration number:**

ISRCTN07689702; pre-results.

## Introduction

There has been considerable investment in studying and improving patient safety but progress is slow.^1–3^ Patient safety incident (PSI) reporting systems are well established, but significant problems remain including their accuracy in identifying and measuring harm,^4^ their cost^5^ and their effectiveness in supporting organisational learning.^6–9^ Alternative and complementary approaches to gathering intelligence about safety and using this information to stimulate change should be considered, and there is growing evidence that patients and their families may fulfil a significant role here.^10^

In recent years, the attention given to the role of the patient in patient safety has increased. Researchers and policymakers alike have argued that ‘there is considerable scope for [patients] to play an active part’ in ensuring that their care is safe and appropriate.^11^ Indeed, we now know that patients will provide comments on the quality and safety of care using their own experiences and can offer considerable detail about specific problems that might be missed in a staff report. For example, patient reports of safety events or experiences are often not expressed in the limited clinical ‘language’ of safety, which can provide services with richer contextual details that may be useful for both understanding the nature of the problem and identifying potential solutions for preventing reoccurrence.^12–16^ Further, emergent findings indicate that while patients may be reluctant to actively volunteer information about safety incidents, if prompted they are able and willing to do so.^17^

The Berwick Report^18^ commissioned by the UK government following an extensive inquiry into poor standards of quality and safety in a large UK acute hospital^19^ proposed a series of recommendations, including a renewed commitment to organisational learning and meaningful patient involvement at all levels of healthcare. In line with this, over the past 5 years we have developed novel approaches to enable patients to provide feedback on the safety of care.^17 20 21^ The Patient Reporting and Action for a Safe Environment (PRASE) intervention allows patients to anonymously report safety concerns using a theory-based and evidence-based reporting instrument. The process and feasibility of collecting information from patients about these safety concerns is described elsewhere.^21 22^ As part of a previously published large cluster randomised controlled trial across 33 wards in five hospitals (trial registration ISRCTN07689702),^23^ we collected information from inpatients about their safety concerns at three time points over a 12-month study period. Here we use these data to explore if patient feedback could support health services to measure and improve the quality and safety of care by addressing the following research questions:

1. What concerns about safety do hospital patients report?
2. How do patients make sense of and categorise these safety concerns?
3. What is the incidence and nature of PSIs experienced by this sample of patients?

## Methods

### Sample and design

Data reported here were collected using a short set of previously validated survey questions for inpatients,^17^ administered by research staff at their bedside during their hospital stay. Data collection proceeded between May 2013 and September 2014 as part of a multicentre, waitlist design, cluster randomised controlled trial, conducted in 33 hospital wards across three NHS Trusts (five hospital sites) in the north of England. This trial was designed to assess the efficacy of the PRASE intervention, co-designed with patients and hospital staff, the detail of which is reported elsewhere.^22 23^ In total, 15 medical wards and 18 surgical wards agreed to host the study across the three NHS Trusts (21 mixed gender, 6 female, 6 male).

### Procedure

Prior to undertaking data collection, all researchers were issued with a handbook and underwent a full day of training, which included (i) an outline of the research, (ii) an overview of the human factors involved in patient safety, (iii) the consent process, (iv) how to respond to and record a patient incident report and (v) how to use the safety netting protocol should a patient report a serious event that required escalation. They also underwent scenario training and were given an opportunity to familiarise themselves with the measurement tools using the computer software previously developed.^21^ All researchers who were new to the project shadowed and were observed by the team who developed PRASE, prior to interviewing patients alone.

Patients were eligible for participation if they were aged ≥16, able to give informed consent, with a minimum period of 4 hours on the ward. Patients were excluded if they were too ill or distressed to take part, had already taken part in the study within the previous month or were non-English or non-Urdu-speaking patients. Feedback was elicited at any point within a patient’s stay, after a minimum of 4 hours on the ward. Written consent was obtained from all patients. A witnessed consent process was available for those who were happy to participate, but unable to sign the written consent form due to poor literacy or visual impairment, with the consent process witnessed by a second member of the research team. We did not allow responses from proxies or surrogates within this study. Participants were asked to provide basic demographic information regarding age, gender, ethnicity (self-determined), time of present admission and the number of admissions over the previous 5-year period. Participating patients were asked the question, ‘Do you want to tell us something that has concerned you about your care?’. Where patients did wish to report a concern, responses were directly inputted into a tablet computer by a member of the research team^21–23^ using the following prompts:

1. Please tell us what happened with your concern or experience in as much detail as you can.
2. Why do you feel this was a safety concern for you?
3. What do you think could be done to stop this from happening again to you or other patients in the future?

The collective responses to these questions were regarded as one patient incident report and were the unit of analysis for this study.

### Analysis

These data were analysed in two stages. First, patient volunteers were supported by researchers to develop meaningful categories for the patient feedback that reflected the patient perspective, with all patient incident reports then sorted into these categories. Second, all patient incident reports underwent a two-stage clinical review process for the identification of PSIs, with incidence of PSIs documented across the categories.

#### Patient research volunteers recruitment and sample

A patient volunteer panel was recruited through open advertisements. Interested volunteers were provided with information about the project, what was being asked of them, the time commitment and the remuneration available. All potential volunteers (n=10) with relevant experience of healthcare were selected to take part (9 out of 10) and eight attended. Of those attending, seven were female and one male, with a mean age of 59 years (range 44–71). Four reported having a disability.

#### Patient representative categorisation of patient incident reports

Volunteers attended seven meetings between September 2015 and February 2016. Between five and seven volunteers were in attendance at each meeting. All meetings were facilitated by two of the research team (CR and SM). In the initial meeting, volunteers were provided with information about how the patient incident reports had been elicited. They were then presented with a randomly selected 20% of the sample of anonymised patient incident reports (n=231) and asked to group them together into categories, creating the categories inductively without reference to preconceptions or theories.^24^ Each patient incident report was read out by a facilitator (CR or SM) (one of the volunteers was registered as blind) and then discussed within the group, with consensus about the categorisation being reached through discussion. This was an iterative process, the categories and definitions evolved throughout the meetings. Categories were reviewed and definitions were agreed, one category was eliminated and the patient incident reports moved to other categories. The volunteers then worked through the rest of the patient incident reports over five meetings. Our approach to the categorisation exercise with the volunteers was to be supportive and collaborative, an approach that was based on a previous published study that worked with non-clinical representatives in taxonomy development.^24^ To this end, it was not felt appropriate to undertake formal inter-rater reliability estimates.

#### Classifying the patient incident reports as PSIs

To address the third research question, patient incident reports underwent a two-stage review process by health professionals.^25 26^ While there are a number of different approaches for reviewing documentation for evidence of safety events,^25–28^ it was felt by the research team that this was the most appropriate method to use, given the very structured nature of the prompts eliciting concerns from patients. Stage 1 comprised two clinical researchers individually reviewing all patient incident reports (n=1155) for the presence of a PSI. The nationally accepted definition for PSIs was used: ‘Any unintended or unexpected incident which could have or did lead to harm for one or more patients receiving NHS care’.^29^ Any patient incident report that was judged to meet this definition by either reviewer was sent to second-stage medical review, with a total of 603 patient incident reports (52%) proceeding to the second stage. To create a consistent approach to the classification process, three doctors (representing respiratory medicine, obstetrics and gynaecology, elderly medicine) first independently reviewed a randomly selected sample of 50 patient incident reports before coming together to discuss and reach consensus about what constituted a PSI. The remaining patient incident reports (553) were then divided between the medical reviewers for second-stage review of the presence of a PSI. Following an approach previously used by the research team,^17^ reports classified as a PSI were then rated against the standard risk indices of (i) preventability (using a four-point scale: 1=‘definitely not preventable’ through to 4=‘definitely preventable’^19^; and (ii) severity (using a five-point scale: 1=‘negligible’ through to 5=‘catastrophic’). Severity was rated as the actual, rather than the potential, severity of the PSI, with preventability concerned with the event rather than associated harm.

## Results

### Sample

Of the 2471 patients recruited in the trial, 579 patients (23%) provided a total of 1053 patient incident reports. In 83 patient incident reports, more than one safety event was identified, giving an overall total of 1155 patient incident reports for analysis. Table 1 presents the demographics for the study sample.

**Table 1:** Sample demographics

	Total sample	Subsample of patients providing one or more patient incident reports
*N*	2471	579
Age		
Mean (SD)	60 (18.3)	56 (18.0)
Median (min, max)	63 (16–103)	58 (16–91)
Missing, n (%)	16 (0.6)	4 (0.7)
Gender		
Female, n (%)	1155 (46.7)	303 (52.3)
Male, (%)	1289 (52.2)	272 (47.0)
Missing, n (%)	27 (1.1)	4 (0.7)
Ethnicity, *n* (%)		
White British	2295 (93)	547 (94.5)
South Asian	51 (2)	8 (1)
Other ethnic origin	101 (4)	21 (4)
Missing	24 (1)	3 (0.5)
Number of inpatient admissions over the previous 5 years		
Mean (SD)	2 (5.9)	3 (8.1)
Median (min, max)	1 (0–100)	1 (0–100)
Time in hospital to date (in days)		
Mean (SD)	7 (12.0)	7 (10.2)
Median (min, max)	3 (0–167)	4 (0–95)
Ward specialty		
Surgical specialities/patients recruited (%)	18 wards, n=1481 (60)	390 (67)
Medical specialities/patients recruited (%)	15 wards, n=990 (40)	189 (33)

### Research question 1: what concerns about safety do hospital patients report?

The patient incident reports were sorted into 14 categories (online supplementary figure and table 2). ‘Communication’ was the most frequently occurring category with a total of 251 patient incident reports (22%). Three types of communication issues were identified by patients: staff to patient, staff to staff and patient to staff, with the first of these being the most frequently cited safety concern. Staff issues, such as availability of staff, insufficient staffing or indicators of this (eg, buzzers not being answered), were the second most common safety concern representing 13% of the total patient incident reports (n=153). Third was ‘environment’, including issues relating to noise at night, lighting levels, the ward layout, and so on (141, 12%). Issues of ‘compassion/dignity/privacy/respect’ (135, 12%) were also mentioned frequently by patients.

10.1136/bmjqs-2017-006974.supp1 Supplementary file 1

**Table 2:** Patient-derived safety categories by rank, with definitions and examples

Rank	Category	Patient incident reports, n (%)	Examples of patient incident reports that were not classified as patient safety incidents	Example of patient incident reports classified as patient safety incidents
1	Communication *All* Staff to patient Staff to staff Patient to staff	251 (21.7%)	Staff to patient: Patient went for a scan, X-ray, without any warning or information about why the scan was being done. Staff to staff: One nurse said I could go home after an X-ray and a possible dressing change, the doctor said that I would go home as I was and have the X-ray as an outpatient and go home as I am. Patient to staff: Patient concerned about fire procedures in case a fire started. The patient is partially sighted and felt that in an emergency he would be left. The side bars to the bed are up and he felt that he would be unable to get out of the bed. Fire procedures have not been explained to him.	Staff to patient: Patient feels there has been some confusion about when they were going to surgery and when they could eat. This has led to them not being able to eat properly for up to 2.5 days at one point. Staff to staff: On some occasions, the night staff do not seem to be aware of the patient’s medication, he has to let them know what he should be taking. Patient to staff: When patient overhears staff talking they assume she is not in pain, but she is, they didn’t ask her and assume that as they have given her painkillers they have worked.
		145 (12.6%)		
		93 (8%)		
		13 (1.1%)		
2	Staff issues For example, staff availability, insufficient staff, not prioritising, buzzers not answered, avoiding work	153 (13.2%)	Staff seemed overworked and that meant things sometimes were not done on time.	I had a cannula replacement to be done and instead of it being done at 18:00 when asked I was knocked awake at 02:00 for it to be done. Because there was no trained doctor available.
3	Environment For example, light at night, noise from staff/other patients/equipment, fixtures and fittings, general cleanliness, temperature, loneliness (single rooms), missing equipment	141 (12.2%)	Night staff were noisy—talking loudly, shoes—made it difficult to fall asleep. Doors slam.	The bath is very difficult to access because the side is high. I have to use a step which is dangerous when you are wet.
4	Compassion/dignity/privacy/respect For example, inappropriate conduct from staff, confidentiality breaches, poor staff attitude, patients not being treated with dignity, overheard private conversation	135 (11.6%)	Consultants need to remember that there is a patient as well as an illness.	On a night time, struggled getting out of bed. The call bell was put out of reach, and couldn’t get out of bed and second night the call bell was again out of reach and the patient had to crawl out of bed as the side railings were put up, and when patient got out the staff were sat around eating take away.
5	Medication issues For example, medicine unavailable, late medication, missed or wrong medication	114 (9.9%)	Patient stated that there were delays in getting anti-sickness tablets following admission. Patient needed them before meals but on several occasions got them too late.	Patient was almost given anticoagulant twice in same day, but stopped nurse and told them that he had already had it.
6	Delay For example, feeling like one is waiting too long, having to reschedule/postpone, results not available, waiting for treatment/investigation, equipment not available	102 (8.3%)	It takes a long time for the discharge process to happen once discharge has been decided, up to 8 hours.	When I came in they told me I would be able to have an operation on Thursday, then changed until Saturday then Sunday and now not till Thursday.
7	Staff training For example, staff not knowing how to do things, staff not trained how to do things, misdiagnosis	63 (5.5%)	Electrically operated bed: staff don’t know how to use it, patient knows more.	Another patient in the bay has dementia and has been aggressive towards the staff. He was punching and grabbing the staff and they were unable to get out of his grasp. A senior nurse came and showed the staff what to do in this situation.
8	Food and drink/nutrition For example, timing of meals and drinks, communication about food requirements, frequent starving for surgery, specific dietary needs, lack of help with eating	54 (4.7%)	Patient has been unable to eat anything but breakfast for the last three days as the food is unpleasant.	Patient opposite cannot communicate her needs or feed herself. She has not had a drink today or had more than two spoons of soup to eat. I felt that the staff feeding her gave up too soon.
9	Ward management For example, lack of consistency, lack of overview, not adhering to a standard, inappropriate ward for patient, missing/mislaid documents	44 (3.8%)	Went to nurses desk to ask a question, and while I was gone they put someone else in my cubicle. Had to sit on chairs for around 30 min—until porter took me to the surgical assessment ward.	File with the notes has been missing from the bedside for the morning—the staff nurse was looking for it, and now the doctor is also looking for it.
10	Equipment and systems failure For example, ward/medical equipment, systems not working or failing	32 (2.8%)	The night lights have been flickering, it gives me a headache.	PCAS machine for pain relief stopped working, two further machines were not working either and they had to get a technician out to come and repair it.
11	Infection risk For example, spills not cleaned up, poor hygiene practice, full bedpans, inappropriate glove use, dirty facilities (visible bodily fluids)	27 (2.3%)	Bed pans (full) had been left in the toilets for hours and smell from one of them was horrendous.	Patient went into toilet last night and noticed faeces on toilet roll holder, she told a member of staff at the time but it was still there following day.
12	Health and safety For example, slips/trips/ falls, clutter, space, safety and security, safeguarding, supervision	27 (2.3%)	Patient described a patient in the same bay who has dementia and causes some disruption—trying to get in bed with another patient. Sometimes patient woke up and saw her standing over her and it is quite scary, especially as quite poorly herself.	There are fire doors at the end of the bay that lead directly outside. In the middle of the night an elderly confused man walked out of them with his Zimmer frame.
13	Repeat procedure/complication For example, something that should not have needed to be done again, unexpected complication	11 (1%)	Came in about 6 weeks ago with abdominal pain—went for scan. Said all fine and sent me home even though I was still not well, as was sick at home. Then readmitted for same problem and now finally having treatment.	I had a blood test that went missing in the laboratory, it had to be repeated.
14	Not a concern	1 (0.09%)	The patient was moved from an orthopaedic ward to a surgical ward because of bed shortages. The move was made once her care needs were appropriate for being moved to a non-specialist area. A nurse from the trauma ward will have to attend to make adjustments to knee brace. (Not a concern, the patient was involved in the decision to move her.)	

PCAS: Patient-Controlled Analgesia System.

### Research question 2: how do patients make sense of and categorise these safety concerns?

The definitions of the categories as agreed by the volunteers and research team are shown in table 2. Each category definition is illustrated with examples.

### Research question 3: what is the incidence and nature of PSIs experienced by this sample of patients?

Across the two-stage review process, 406 of the total 1155 patient incident reports (35%) were classified by clinicians as a PSI according to the standard definition. Of the 2471 patients recruited to the study in total, 264 reported one or more PSIs (10.68%), meaning that 1 in 10 patients identified a PSI during their inpatient stay. Eighty-seven individuals (3.52% of the total sample) reported more than one PSI (range=2–7; median=2). Table 2 provides examples of patient incident reports from each patient-derived category reported by patients that were and were not classified as a PSI.

**Figure 1:**
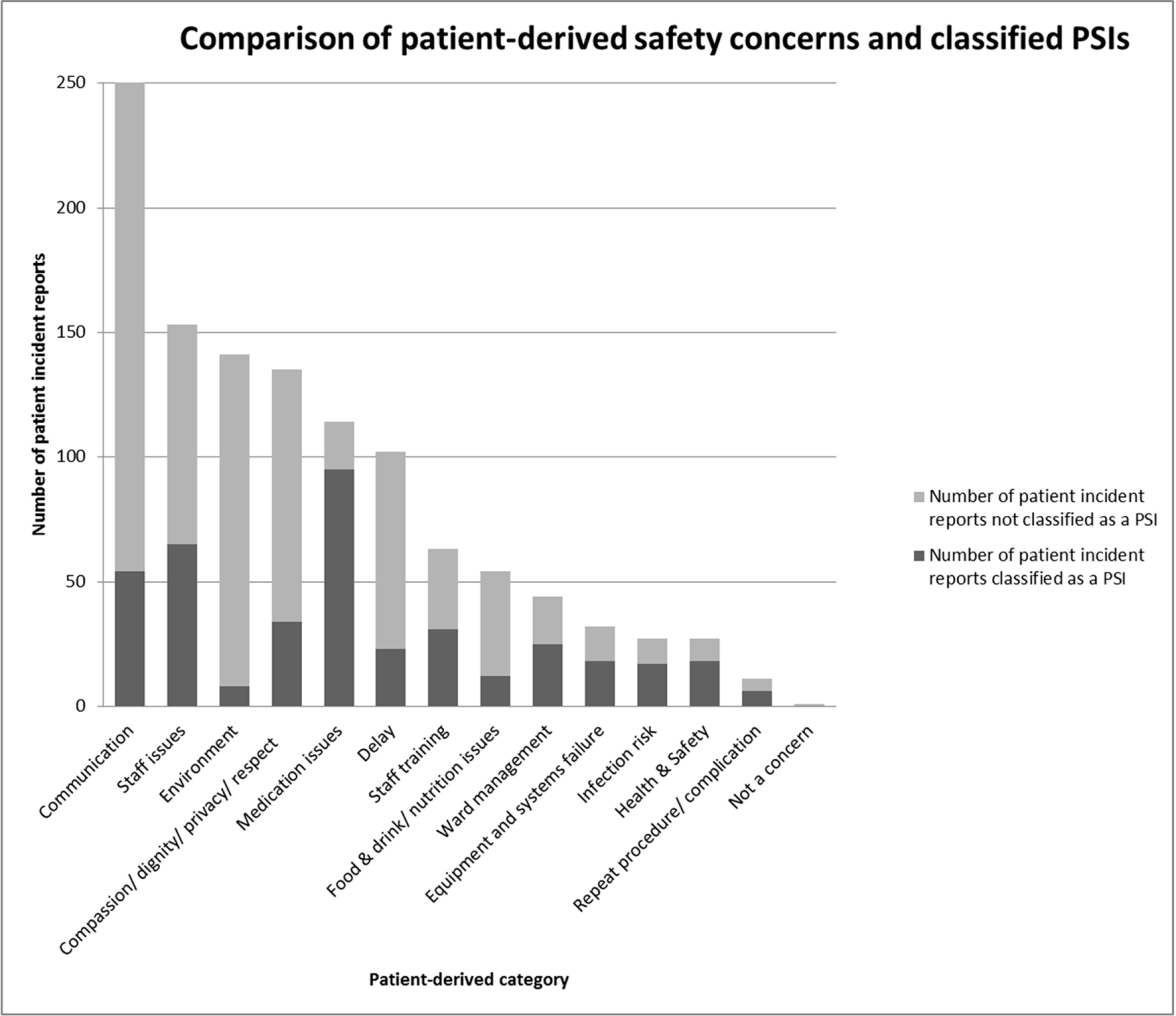
Comparison of patient safety concerns and classified patient safety incidents (PSIs), by category.

The number of patient incident reports that were classified as PSIs in each patient-derived category is presented in figure 1 and table 3. For the first stage of classification, inter-rater reliability between the reviewers was good, with 62% of the initial classifications agreed on. Of those categorised as a PSI, the inter-rater agreement for the other ratings undertaken was good: (i) 95% for likelihood of preventability (when grouped as ‘definitely or probably preventable’ and ‘definitely or probably not preventable’) and (ii) 100% for the degree of severity (when grouped as ‘negligible, minor or moderate’ and ‘major or catastrophic’). Table 4 provides further detail of these assessments.

**Table 3:** Frequency and percentages of classified patient safety incidents (PSIs) by patient-derived safety category

Category	PSIs (n)	PSIs within category as a percentage of total classified PSIs (n, %)	PSIs as a percentage of patient incident reports within category (%)	PSIs as a percentage of the total number of patient incident reports (%)
Communication	54	13	21.5	4.7
Staff issues	65	16	42.5	5.6
Environment	8	2	5.7	0.7
Compassion/dignity/privacy/respect	34	8	25.2	2.9
Medication issues	95	23	83.3	8.2
Delay	23	6	22.5	2.0
Staff training	31	8	49.2	2.7
Food and drink/nutrition	12	3	22.2	1.0
Ward management	25	6	56.8	2.2
Equipment and systems failure	18	4	56.3	1.6
Infection risk	17	4	63.0	1.5
Health and safety	18	4	66.7	1.6
Repeat procedure/complication	6	2	54.5	0.5
Not a concern	0	0	0	0

**Table 4:** Assessed severity and preventability of patient safety incidents, and percentage agreement between reviewers

Actual Harm			Avoidability		
	Frequency	% agreement		Frequency	% agreement
Negligible*	186	45.81	Definitely preventable*	215	52.96
Minor*	44	10.84	Probably preventable*	47	11.57
Moderate*	4	0.99	Probably not preventable*	1	0.25
Major*	1	0.25	Definitely not preventable*	0	0
Catastrophic*	0	0			
Total agreement		58	Total agreement		65
Negligible, minor, moderate*	405	99.75	Definitely preventable, probably preventable*	384	94.58
Major, catastrophic*	1	0.25	Probably not preventable, definitely not preventable*	1	0.25
Total agreement when dichotomised		100	Total agreement when dichotomised		95

*Figures presented represent those for which there was agreement between reviewers, with the sum therefore not matching the total number of classified PSIs.

The degree to which patient-reported incidents were classified as PSIs varied (table 3). In the communications category, despite having the largest total of patient-reported incidents, only 54 of these (21.5%) were classified as PSIs following clinical review. Other categories with the largest difference (<50%) between the number of patient reports and those classified as PSIs were ‘staff training’ (49.2%), ‘staff issues’ (42.5%), ‘compassion, dignity and respect’ (25.2%), ‘delay’ (22.5%), ‘food, drink and nutrition’ (22.2%), ‘environment’ (5.7%) and ‘not a concern’ (0%). Those categories where there was found to be closer alignment between the number of patient reports and the number of classified PSIs (>50%) were ‘medication issues’ (83.3%), ‘ward management’ (56.8%), ‘equipment and systems failure’ (56.3%), ‘infection risk’ (63%), ‘health and safety’ (66.7%) and ‘repeat procedure/complication’ (54.5%).

Most classified PSIs were rated as ‘negligible, minor or moderate’ in terms of severity (99%). However, the majority of PSIs were also rated as ‘probably or definitely avoidable’ (90%).

## Discussion

The data reveal that patients are an important source of safety reporting, with 1 in 10 reporting a safety concern that meets the clinical definition of a PSI. While this rate may appear strikingly similar to long-held estimates of patient harm in hospitals,^4 30^ our research team has previously demonstrated that patient-reported safety events rarely overlap with events identified through other error detection methods.^31^ This suggests that our current methods of safety measurement in hospital settings (eg, case note review and staff incident reporting) may underestimate the level of PSIs. A large observational study of adverse events examining a range of safety data^32^ supports this, having found that 17.7% of patients experienced a serious harm event. Further, what patients tell us appears to be both concordant with knowledge gained from other existing processes but also provides a unique perspective by capturing concerns that are important to patients but overlooked by clinical reporting systems.^31 33^ In our study, 65% of the concerns expressed by patients in this study would not traditionally be classified as PSIs including concerns about physical comfort (eg, noise and light levels, food), fear (eg, of other patients), uncertainty (eg, about when discharge is happening) and delays (eg, in procedure). Patients may sometimes be misplaced in their fears or have forgotten the explanation of a treatment plan. However, many patient-reported safety concerns provide valuable personal insight into how care is experienced by the patient, and therefore what could be done to improve both patient safety and patient experience.

The safety concerns that patients report may be ignored by our current error detection methods (such as communication, delays in care processes), and yet they are known contributory factors to future safety events,^34^ making patient-reported safety concerns a possible leading measure of safety. It could also be argued that the information provided by patients is less biased in its content than incident reporting systems and less time consuming to collect than case note review. Taken collectively, these advantages over existing safety measures in hospitals present strong arguments for the positioning of patient feedback on safety as a key indicator of safety. In fact, given the advantages, one might even go as far as to propose that patient feedback could be used as one of the primary mechanisms for gathering safety intelligence, with the caveat that this approach is used as an improvement tool by ward/unit/practice teams rather than as an external regulatory or validation tool.

While there has been a number of important studies published over the past decade gathering the patient perspective of safety,^12–16^ this is, to our knowledge, the largest study of its kind. Further, our approach—to seek to understand and categorise the patients’ reported safety concerns with patient representatives—is novel. One of the recent criticisms of the lack of progress in using patient feedback to support patient safety improvements is that the information does not necessarily fit within our current professionally developed systems for managing safety and clinical risk.^10^ Part of this problem is due to the difficulty in incorporating patient feedback into our current methods of capturing safety data. In recent work (funded by the Health Foundation), hospital volunteers have worked with ward teams to collect this patient safety feedback from patients and to make improvement plans. There is huge potential for this approach to support local learning and improvement and move beyond the current focus on the collection of safety data alone.

The science and practice of patient safety is arguably undergoing a paradigm shift, with a move towards focusing less on past harm, and more on understanding what supports safe care and resilience in our services and systems.^35 36^ Gathering patient feedback about safety supports this in two main ways. First, through gathering patient-reported safety concerns, healthcare organisations may gain a unique insight into the ‘little’ things that are suboptimal in safety terms, but do not cause harm—information that is often overlooked by other ‘error detection’ methods. Our finding that a majority of patient-reported PSIs are classified as ‘negligible’ would suggest that patients are perfectly positioned as a source of these leading indicators of safety. By focusing attention on the combination of these smaller, more frequent events, collecting and acting on patients’ safety concerns may facilitate upstream changes that support the creation of an environment where more things go right. Second, through gathering safety concerns systematically, patients and their families can provide information about perceived safety that can provide insight quickly for those managing services. Such real-time insight potentially allows services to make small adjustments to care delivery, as well as aggregating data over time to understand longer-term problems, and build resilience in our services and systems.

To realise the potential benefits of gathering feedback from patients, however, depends in no small part on the ability of services to embed these approaches within their current systems and resources, and act on the data that arises from this sustained activity. The first problem has been explored tentatively by further work undertaken by our research team which sought to understand if patient feedback about safety could be gathered by trained hospital volunteers using tablet computers. Early findings from this work suggest that such an approach is acceptable, feasible and gives rise to data that can be used by ward staff to engage in service improvement.^37^ The second problem—staff acting on patient feedback to improve services and the safety of care—arguably represents a wider and more troublesome issue for the health service improvement. As part of the randomised controlled trial from which the data presented within this paper were drawn,^23^ it was demonstrated that through facilitated action planning ward staff were able to use patient feedback to make changes to services.^38^ However, there are issues that are particular to using patient feedback for improvement, for example, the credence given to the patient perspective of safety, which may undermine the process of acting on this feedback.^39^ Further, while patient experience is widely gathered^40^ and valued at a policy level,^41^ there is little evidence that these data are used for quality improvement.^40 42^ It is likely that with respect to patient feedback about safety this issue will be amplified, and that health services will struggle to incorporate patient feedback about safety within their current mechanisms for measuring, monitoring and managing risk.^10 43^ It will be important, therefore, for the research and healthcare communities to consider how to both create space for staff to consider and act on patient feedback, as well as meaningfully integrate such feedback into their prospective management of patient safety.^10 40^

This study has a number of limitations. Patients in this study were asked to describe concerns about their care, rather than specifically about the safety of their care. However, we have found and reported elsewhere^17^ that patients may be unsure about what is meant by safety and have a less expansive definition of safety from that used in this paper. Therefore, the omission of the word safety in the question we posed was deliberate and recognised that some, although not all, concerns would be classified as relating specifically to safety. A further issue relates to the process of classifying patient reports as PSIs. All judgements about the nature and degree of harm are made on the basis of patient feedback alone, with no reference to further clinical information. It is therefore possible that this either overestimates or underestimates the number of classified PSIs within our sample. A final limitation relates to the inter-rater reliability estimates for both the categorisation of patient incident reports by patient volunteers and the classification of these reports as PSIs by medical reviewers. No estimates were collected as to the degree of variation between volunteers in the categorisation exercise. However, given the nature of the categorisation process, and the need to facilitate the process with those not trained in research methods, it was felt that a collaborative, consensus building approach was more appropriate. Further, we only calculated inter-rater agreement figures between the medical reviewers for a random sample of 50 patient incident reports in the first exercise of classifying the patient incident reports as PSIs. This was due to the volume of reports gathered from patients (1155). However, of those classified as a PSI, all reports were assessed for severity and the likelihood of avoidability, for which the level of agreement was large when grouped meaningfully.

In conclusion, our findings suggest that patients can provide important insight about safety that complements our existing error detection methods. Patients report a similar frequency of PSIs to clinical and epidemiological reports. However, they provide a unique and distinctive perspective about hospital safety that encompasses a wider understanding about patient experiences that are not captured in current reporting systems. As such, gathering and acting on the patient perspective of safety has the potential to help build resilience in care processes and improve future safety performance among clinical teams and across organisations.
