# Supplementary figures and images for: What can patients tell us about the quality and safety of hospital care? Findings from a UK multicentre survey study
Source: BMJ Qual Saf. 2018 Mar 15;27(9):673–82. doi: 10.1136/bmjqs-2017-006974 (PMC6109253; doi:10.1136/bmjqs-2017-006974)

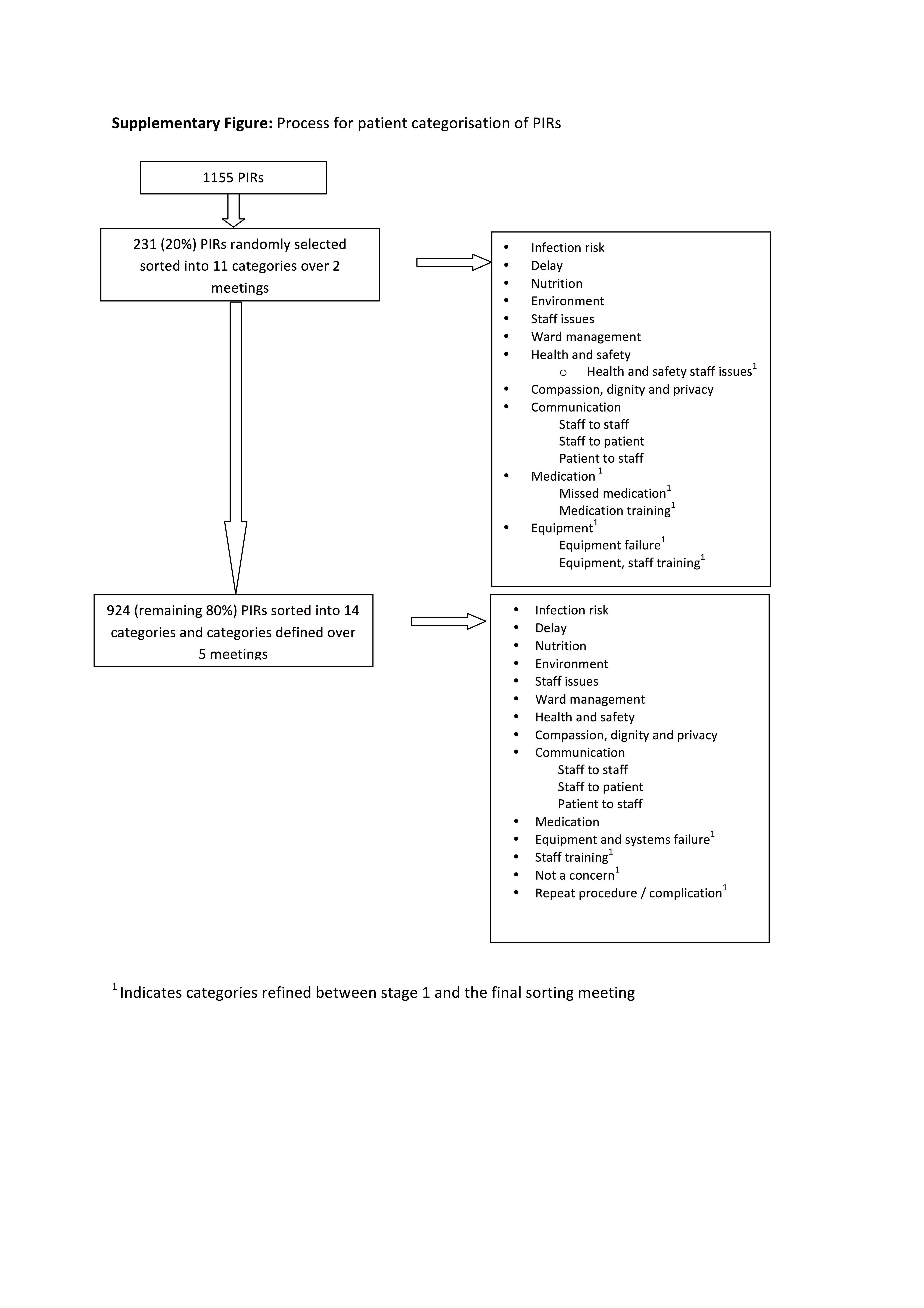

Supplement: Supplementary file 1 [file bmjqs-2017-006974supp001.jpg]
